# Investigation of the Influence of Reduced Graphene Oxide Flakes in the Dielectric on Surface Characteristics and Material Removal Rate in EDM

**DOI:** 10.3390/ma12060943

**Published:** 2019-03-21

**Authors:** Rafał Świercz, Dorota Oniszczuk-Świercz

**Affiliations:** Institute of Manufacturing Technology, Warsaw University of Technology, 00-661 Warsaw, Poland; doo@meil.pw.edu.pl

**Keywords:** EDM, RGO, power-mixed, surface roughness, recast layer, reduced graphene oxide, material removal rate, electrical discharge machining

## Abstract

Electrical discharge machining (EDM) is an advanced technology used to manufacture difficult-to-cut conductive materials. However, the surface layer properties after EDM require additional finishing operations in many cases. Therefore, new methods implemented in EDM are being developed to improve surface characteristics and the material removal rate. This paper presents new research about improving the surface integrity of 55NiCrMoV7 tool steel by using reduced graphene oxide (RGO) flakes in the dielectric. The main goal of the research was to investigate the influence of RGO flakes in the dielectric on electrical discharge propagation and heat dissipation in the gap. The investigation of the influence of discharge current *I* and pulse time *t*_on_ during EDM with RGO flakes in the dielectric was carried out using response surface methodology. Furthermore, the surface texture properties and metallographic structure after EDM with RGO in the dielectric and conventional EDM were investigated and described. The obtained results indicate that using RGO flakes in the dielectric leads to a decreased surface roughness and recast layer thickness with an increased material removal rate (MRR). The presence of RGO flakes in the dielectric reduced the breakdown voltage and allowed several discharges to occur during one pulse. The dispersion of the discharge caused a decrease in the energy delivered to the workpiece. In terms of the finishing EDM parameters, there was a 460% reduction in roughness *Ra* with a uniform distribution of the recast layer on the surface, and a slight increase in MRR (12%) was obtained.

## 1. Introduction

Recent requirements in manufacturing processes have led to the production of complex shape parts from difficult-to-cut materials. One of the main nonconventional technologies that is widely used to manufacture conductive parts regardless of their hardness is electrical discharge machining (EDM). EDM has been widely used for the manufacturing of dies and molds and of aerospace parts [1,2,3,4]. The mechanism of material removal is the result of electrical discharge, which causes local melting and evaporation of a small volume of material. Local rapid thermal processes result in changes to the external surface layers of the material. An analysis of the metallographic structure indicates that, in tool steel after EDM, typical layers with different properties from the core material can be observed: a recast layer, a heat affect zone, and a tempered layer [5,6]. The parameters of electrical discharge (discharge voltage, discharge current, and time pulse) determine the value of discharge energy. These parameters directly affect the surface integrity and material removal rate (MRR) [7,8,9,10]. The processing condition, type of dielectric, and electrode and workpiece material also have a strong influence on the quality of parts after EDM [11,12,13,14]. A number of researchers have worked in the area of modeling [15,16,17] and optimization of EDM [18,19,20,21,22]. The presented results indicate that the discharge stability and gap condition have key roles in the surface quality and MRR. 

The surface properties after EDM in many cases require additional finishing operations [23,24,25,26] or coatings [27,28,29] for industrial application. This leads to an increased production cost. Therefore, new methods are being developed to improve the quality of the surface and material removal rate [30,31].

In order to improve surface integrity and material removal rate, a number of researchers have investigated the influence of additional particles in the dielectric on EDM. The use of additional particles in the gap changes the ignition and discharge process. In conventional EDM, the probability of discharge in a specific location depends on the value of the breakdown voltage of the dielectric in the gap. A high voltage initiates the ignition of the discharge between the electrode immersed in the dielectric. After exceeding the dielectric breakdown strength, a plasma channel is formed. Around the plasma channel, a bubble gas is created, which is filled with ions and parts of melted material of the workpiece and the electrode. At the end of the discharge, the bubble gas and plasma channel implosively collapse. The melted material is thrown away to the gap and rapidly cooled down by the dielectric. The molten material is resolidified into hundreds of spherical particles. Depending on the discharge energy, the size of debris varies and can reach several micrometers. The debris and bubble gas are removed from the gap by the flushing dielectric. The conditions in the gap stabilize during a time interval. Another discharge takes place in a random place. The overlapping of discharge craters generates a specific geometric structure of the surface. The craters should have similar shape and depth to ensure uniform properties of the surface. 

One of the major effects influencing the uneven discharge location, shape, and depth of craters is the presence of debris and bubble gas in the gap [32]. Murray et al. [33] indicated that a local concentration of debris may lead to the occurrence of discharges in the same place. Research carried out by Hayakawa et al. [34] showed that debris can be scattered near the boundary of discharged bubbles. Ayesta et al. [35] pointed out that ineffective removal of debris from the gap leads to short-circuiting and arc discharges. Research carried out by Tanjilul et al. [36] and Wang et al. [37] showed that, in the case of EDM drilling, the removal of debris from the gap has a strong influence on the material removal rate and surface roughness. 

Effective removal of debris has a key role in stabilizing electrical discharges and the repeatability of the process. Research carried out by Talla et al. [38] and Wu et al. [39] indicated that stable electrical discharges with a lower energy occurred with the use of additional particles in the gap. The presence of conductive particles decreased the dielectric voltage breakdown. The ignition of the discharge took place with an increased gap in relation to conventional EDM. The formation of a bridge can explain this phenomenon by conductive particles in the dielectric, which facilitates ignition. Depending on the material of the additional particles, their size and electrical discharge parameters, surface integrity, and material removal rate will change. Kumar et al. [40] indicated that nanopowder particles in the dielectric gave a better surface finish and higher metal removal rate as compared to conventional EDM. Surekha et al. [41] indicated that in a powder-mixed discharge machining, the parameter with the most influence on the material removal rate was the discharge current, followed by the concentration of conductive particles. Research conducted by Ou et al. [42] showed that using additional particles in the dielectric improved the surface integrity in the manufacturing of titanium alloys with bioactive hydroxyapatite powder. Prakash et al. [43] showed that additional particles of Si powder in the dielectric during machining titanium alloy could reduce the number of microcracks in the surface layer. Sahu et al. [44] reported that SiC particles in the dielectric had a strong influence on the residual stress during the machining of Inconel 718 alloy. The authors indicated that heat flux during discharge was also transferred to the abrasive particles. At the end of discharge, it was balanced by the rapid cooling of the workpiece. This provided relatively less thermal stress than in conventional EDM. Tijo et al. [45] proposed the use of Ti and B4C powder-mixed electrical discharge machining with a coating of Ti-6Al-4V. The results showed the possibility of improving the microhardness of the external layer (up to three times) and a significant reduction in the surface wear rate (up to seven times). Xie et al. [46] indicated that by coating the surface using titanium electrode, additional graphite powder particles in the dielectric caused a uniform thickness of the coated layer with a reduced number of microcracks. The dispersion of the electrical discharge on the graphite powder led to more uniform energy throughout the surface.

From the literature survey, it was noticed that a sufficient amount of work has been carried out on improving surface integrity and material removal rate using powder mixed electric discharge machining (PMEDM). However, the use of reduced graphene oxide flakes in the dielectric is not well described. Due to the properties of reduced graphene oxide flakes (RGO) (high electrical and thermal conductivity) [47,48], ignition of electric discharge and heat dissipation in the gap will be different from the processing in the pure dielectric. Facilitating the initiation of electrical discharge with an increased gap size results in stable electrical discharges with reduced energy.

The main goal of this research was to investigate the influence of RGO flakes in the dielectric on electrical discharge propagation and its influence on the surface roughness, surface layers, and material removal rate. It was expected that changing the discharge propagation would improve the surface roughness and material removal rate and decrease the thickness of the recast layer. To examine this assumption, experimental studies were conducted. An investigation of the influence of the discharge current and pulse time during EDM with RGO flakes in the dielectric was carried out using response surface methodology. Furthermore, the surface texture properties and metallographic structure after EDM with RGO in the dielectric and conventional EDM were investigated and described. In the next stage of the research, the regression equations of the influenced discharge, current, and pulse time on roughness *Ra* and MRR were established for manufacturing in the pure dielectric and with RGO flakes.

## 2. Materials and Methods

In this study, the influence of reduced graphene oxide (RGO) flakes in the dielectric on discharge propagation and heat dissipation in the gap was analyzed. The Charmilles FORM die-skinning EDM machine (GF Solutions, Geneva, Switzerland) was used for the experiment. Heat-treated 55NiCrMoV7 tool steel (55 HRC) was used as a workpiece. The samples had dimensions of 10 × 10 × 3 mm. This material is widely used for forging dies, die inserts, and dies for hydraulic and mechanical presses. Table 1 shows the chemical composition of the steel. A copper electrode with a cross-sectional area of 12 × 12 mm was used in this study, and 108 MP-SE 60 EDM fluid was used as the dielectric. All samples in the tests were ground before EDM machining in order to achieve the same surface roughness properties. The experimental investigation was performed at sinking EDM configuration.

**Figure 1:**
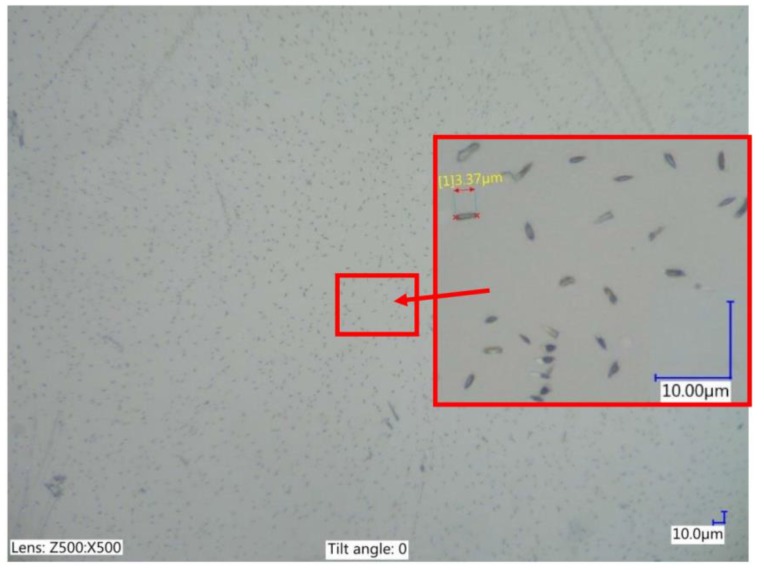
The reduced graphene oxide (RGO) in the dielectric.

Reduced graphene oxide was developed at the Graphene Laboratory of Warsaw University of Technology. In this study, 0.1% RGO (Figure 1) with an average area of 2 µm^2^ was used in the dielectric. The calculation of the average of RGO flakes was conducted with Keyence VHX software (Keyence, Osaka, Japan) based on an analysis of the flakes in the dielectric on a Keyence VHX-6000 digital microscope (Keyence, Osaka, Japan). The properties and method of production of the RGO flakes were described in Reference [49].

Research carried out so far [50] has indicated that stable discharges occur with reduced graphene oxide in dielectric for 0.1% of RGO. Preliminary research indicates that with an increased concentration of RGO in the dielectric, discharge energy must also increase to cause local melting and evaporation of the material. An explanation of this effect can be found in RGO properties. The high electrical conductivity of reduced graphene oxide flakes causes dispersion of electrons. In the case of finishing EDM parameters (discharge current *I* = 1 A, time pulse *t*_on_ = 5 µs) and concentration of RGO in the dielectric, more than 0.5% energy transferred to the surface is not enough to melt and evaporate the material.

Preliminary research has shown that the main influences on the surface roughness and surface layer properties are discharge current *I*_c_ and pulse time *t*_on_. For a constant voltage, these 2 parameters accordingly define the discharge energy, according to Equation (1):

(1) $$E=\int_{0}^{t_{on}}U_{c}\left(t\right)\cdot I_{c}\left(t\right)dt\;\left(\mathrm{mJ}\right),\;$$

Considering the above relationship, a literature review, and the preliminary research, the parameters for EDM roughing, semifinishing, and finishing machining were established. Experimental studies were conducted using the designed experimental methodology. A rotatable design with 5 levels and 2 parameters was used. Table 2 presents the conditions of the experiment.

In order to prevent a concentration of RGO in the dielectric in one place, the working fluid was stirred with a rotating screw during EDM.

The present paper is focused on investigating the influence of reduced graphene oxide (RGO) flakes in the dielectric on the surface roughness, the thickness of the white layer, and the material removal rate. Measurements of the surface roughness after EDM were made using the Taylor Hobson Form Talysurf Series 2 scanning profilometer (Taylor Hobson, Leicester, UK). The roughness parameter *Ra* was measured in 5 sections, and an average value was calculated.

Metallographic surface structure studies were performed on specially prepared samples. Each sample after EDM was cut in half with wire electrical discharge machining perpendicular to the machined surface. Next, the samples were included in the resin and then ground and polished. Micro-etching was performed with nital (5%) to reveal the microstructure of the material. The metallographic structure of the specimens was analyzed with a Nikon Eclipse LV 150 optical microscope (Nikon, Tokyo, Japan), coupled to a NIS-Elements BR 3.0 image analyzer (Nikon, Tokyo, Japan) and Keyence VHX-6000 digital microscope (Keyence, Osaka, Japan).

The material removal rate (MRR) was calculated based on the volume of material removed from the workpiece divided by the machining time:

(2) $$\mathrm{MRR}=\frac{m_{1}-m_{2}}{\rho \;\Delta t}\;\left[\frac{mm^{3}}{min}\right]$$

where *m*_1_ is sample weight before processing, *m*_2_ is sample weight after processing, ρ is specific material density, and Δt is time of manufacturing.

Each sample was weighed before manufacturing on a precision electronic balance (Radwag, Radom, Poland). After the EDM process, the samples were cleaned with compressed air and then weighed again.

During electrical discharge machining, the current and voltage waveforms were measured using an oscilloscope card (NI5133, National Instruments, Austin, TX, USA). The current was measured with a noninductive current sensor. The voltage during the electric discharge was measured with a Tektronix probe (Tektronix UK Ltd., Berkshire, UK). The sampling rate was 100 MS/s, 2-channel registration. An application was developed in the LabView environment that enabled control of the oscilloscope card function. The obtained data were analyzed in DIAdem (National Instruments, Austin, TX, USA).

## 3. Results and Discussion

### 3.1. Analysis of Influence of RGO Flakes in Dielectric on Surface Integrity

In this research, an analysis of the influence of RGO flakes in the dielectric (0.1% concentration) on the discharge propagation and heat dissipation in the gap was conducted. A series of experimental tests was performed according to the design of experimental methodology. The results of the test are presented in Table 3. Roughness *Ra* was in the range of 1.73 µm to 12.29 µm and from 0.37 µm to 7.72 µm, respectively, for manufacturing in the pure dielectric and the dielectric with 0.1% RGO. MRR was in the range of 0.42 mm^3^/min to 26.64 mm^3^/min for EDM in the pure dielectric and 0.64 mm^3^/min to 28.77 mm^3^/min for EDM with 0.1% RGO in the dielectric. The obtained values of roughness *Ra* and MRR corresponded to finishing and roughing machining.

The results show that the use of RGO flakes in the dielectric for finishing EDM parameters (example 1) obtained a 460% reduction in roughness *Ra* with a slight increase (12%) in MRR. In the case of roughing EDM (example 4) with 0.1% RGO flakes in the dielectric, roughness *Ra* decreased 60% and MMR increased 27%.

**Figure 2:**
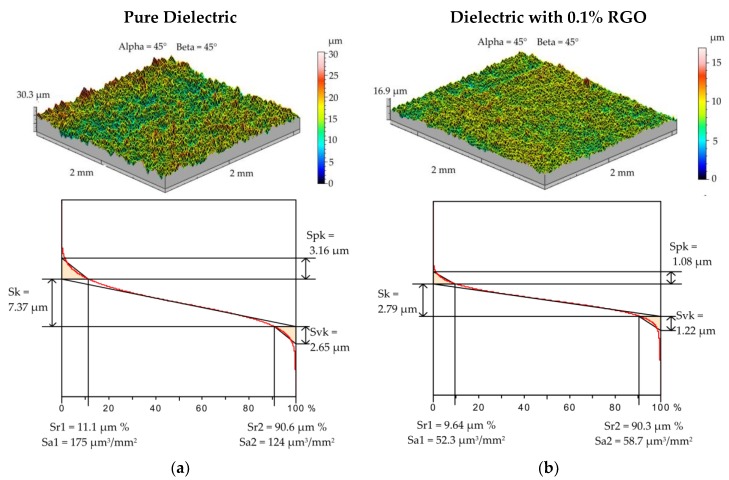
The surface texture and Abbott–Firestone curve after electrical discharge machining (*U_c_* = 25 V, *I* = 2 A, *t*_on_ = 10 µs): (**a**) pure dielectric and (**b**) dielectric with 0.1% RGO.

**Figure 3:**
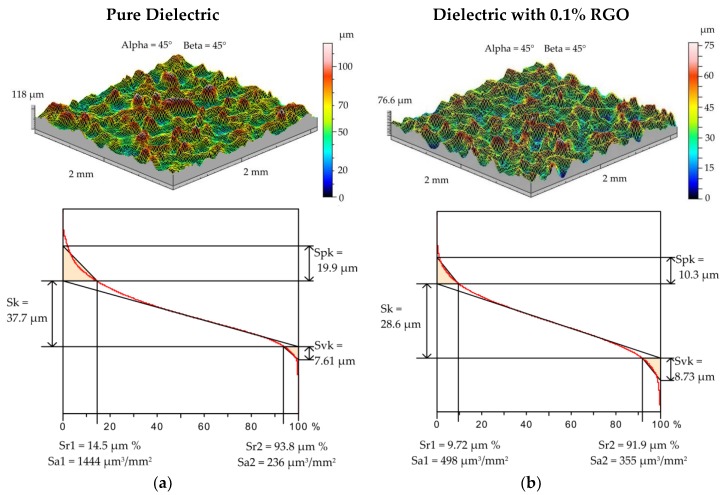
The surface texture and Abbott–Firestone curve after electrical discharge machining (*U_c_* = 25 V, *I* = 13.5 A, *t*_on_ = 145 µs): (**a**) pure dielectric and (**b**) dielectric with 0.1% RGO.

Electrical discharge machining is a thermal process in which material is removed from the workpiece as a result of the melting and evaporation of metal. A series of discharges generates craters that form a surface texture. The properties of surface topography have a strong influence on the fatigue strength and other tribological properties [51,52,53]. For samples manufactured with the smallest and highest values of discharge energy, additional measurements of surface topography were conducted. A 2 × 2 mm area was measured on the profilometer with an x-axis and y-axis discretization step of 10 μm. The tribological properties of the surface can be evaluated by analyzing the load capacity of the surface texture. The load capacity is defined as the ratio of the bearing surface to the total surface and can be described by the Abbott–Firestone curve (Figure 2 and Figure 3). The roughness of valleys *Svk* and lower bearing surface *Sr2* allow the surface lubrication properties to be evaluated. *Svk* and *Sr2* give information about the ability of the fluid to hold through the sliding surfaces. The mean height of the peaks on the core profile *Spk* can provide information about surface resistance to abrasion. The lower the *Spk* value is, the higher the resistance to abrasion. The roughness of core *Sk* determines the depth of the roughness after the initial break-in.

The roughness of the peak and core after finishing EDM (*I* = 13.5 A, t_on_ = 145 µs, *U* = 25 V) in the pure dielectric (Figure 2a) was *Spk* = 3.16 μm and *Sk* = 7.37 μm. The upper bearing surface was *Sr1* = 11%, and the area of elevations filled with material was *Sa1* = 175 μm^3^/mm^2^. Changing the dielectric properties by adding RGO flakes in EDM significantly affected the surface topography and its tribological properties. For the sample after EDM (*I* = 2 A, *t*_on_ =10 µs, *U* = 25 V) in the dielectric with 0.1% RGO, the reduced roughness of peak and core was *Spk* = 1.1 μm and *Sk* = 3.2 μm (Figure 2b). The obtained parameters values are almost 3 times lower than for EDM in the pure dielectric. The roughness of valleys *Svk* = 1.22 µm and area of pits free from the material *Sa2* = 58.7 µm for EDM in 0.1% RGO was reduced by twice as much compared to EDM in the pure dielectric.

For EDM with roughing parameters (*U_c_* = 25 V, *I* = 13.5 A, *t*_on_ = 150 µs) in the dielectric with 0.1% RGO, significant changes in the surface topography in relation to the EDM in the pure dielectric were also observed (Figure 3a,b). A reduction of almost twice as much in both *Spk* and *Sk* parameters was obtained. However, the roughness of valleys *Svk* and lower bearing surface *Sr2* for both samples were similar. For EDM in the pure dielectric, *Svk* = 7.61 μm and *Sr2* = 93.8% relative to *Svk* = 8.73 μm and *Sr2* = 91.9% for EDM with 0.1% RGO in the dielectric. These results show that the depth of craters was similar, but the core and peak were different.

**Figure 4:**
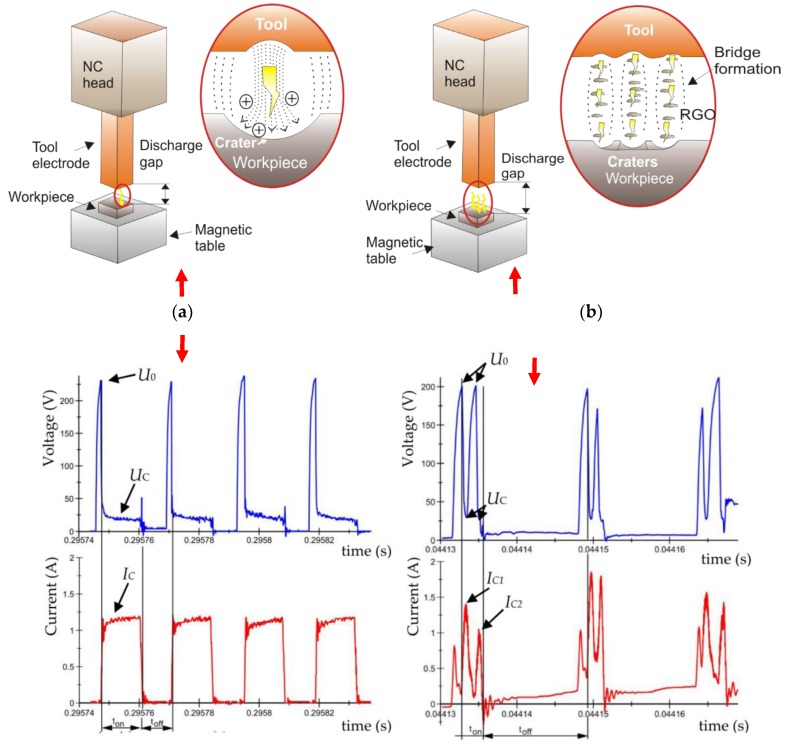
The recorded voltage and current waveforms: EDM fluid dielectric. (**a**) For conventional EDM in discharge time *t*_on_, there is one discharge for the pure dielectric, *U*_0_ = 225 V, *U* = 25 V, *I* = 1.2 A, *t*_on_ = 10 µs, *t*_off_ = 8 µs, and (**b**) for EDM fluid with 0.1% RGO flakes in the dielectric, in one discharge time *t*_on_, there can be several discharges, *U*_0_ = 200 V, *U_C_* = 25 V, *I* = 1.5 A, *t*_on_ = 3 μs, t_off_ = 13 μs.

In conventional EDM, during pulse time, one discharge causes local melting evaporation of the material [54]. During discharge around the plasma channel, bubble gas is formed. At the end of the discharge, the voltage and current drop down. The plasma channel and bubble gas implosively collapse and throw molten material into the gap, which rapidly resolidifies, cooled by the dielectric. Debris and bubble gas are removed from the gap by flushing the dielectric during the time interval (*t*_off_) between next discharge. Efficient removal of the erosion products from the gap prevents a local concentration of discharges, whereas ineffective removal leads to a short circuit. In the case of EDM with additional particles in the dielectric, the physics of the material removal phenomenon are quite different. Using EDM with additional conductive particles in the dielectric contamination facilitates an ignition process with an increased gap size. This allows for better flushing of the gap and removal of debris. The presence of reduced graphene oxide flakes reduces the dielectric breakdown voltage. With the additional particles, several bridges can be form. This can lead to multiple discharges during one pulse. During discharge, the supply voltage *U*_0_ dropped to the discharge voltage *U*_C_. At the same time, the current rose to the discharge current *I*c (Figure 4). In the case of EDM with reduced graphene oxide flakes in the dielectric, an analysis of current voltage waveforms shows that, in one pulse, there can be several discharges. The presented assumption coincides with the study performed by Chao et al. [55] and Gatto et al. [56].

The results indicate that in the case of machining with parameters corresponding to surface finishing and roughing, the use of RGO in the dielectric significantly changes the transport of electrons during electric discharge. Increasing the gap size leads to a decrease in the heat flux and volume of material removed in discharge. During one pulse, the emergence of surface ridges is reduced. Generated craters are shallow, with lower borders. The results indicate similar trends to the results by Shabgard et al. [57] with the use of carbon nanotube.

**Figure 5:**
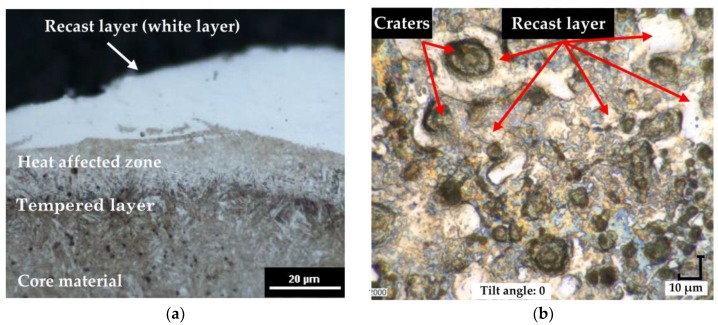
The metallographic structure (**a**) and surface morphology (**b**) of 55NiCrMoV7 tool steel.

The properties of the surface integrity after EDM result mainly from the thermal processes and phase transitions. An observation of the metallographic structure shows three layers (Figure 5a): external recast layer, commonly referred to as a white layer; heat affected zone, which is visible as a bright structure located directly under the recast layer; and tempered layer, which appears in the form of a dark streak. An observation of the surface morphology image (Figure 5b) indicates a nonuniform distribution of the recast layer on the surface.

**Figure 6:**
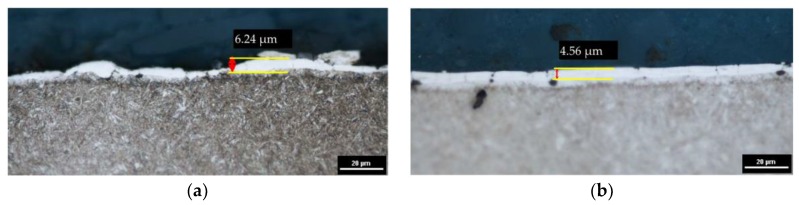
The metallographic structure of 55NiCrMoV7 tool steel after EDM; *Uc* = 25 V, *I* = 1 A, *t*_on_ = 5 µs, *t*_off_ = 5 µs: (**a**) pure dielectric and (**b**) dielectric with 0.1% RGO.

**Figure 7:**
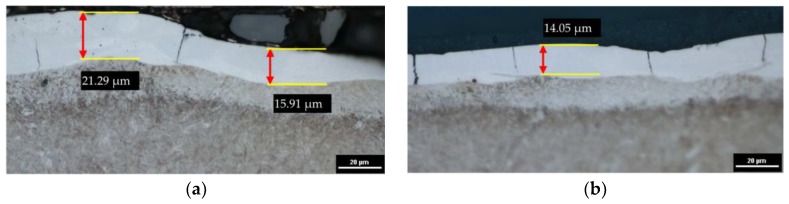
The metallographic structure of 55NiCrMoV7 tool steel after EDM; *Uc* = 25 V, *I* = 8 A *t*_on_ = 75 µs, *t*_off_ = 25 µs: (**a**) pure dielectric and (**b**) dielectric with 0.1% RGO.

**Figure 8:**
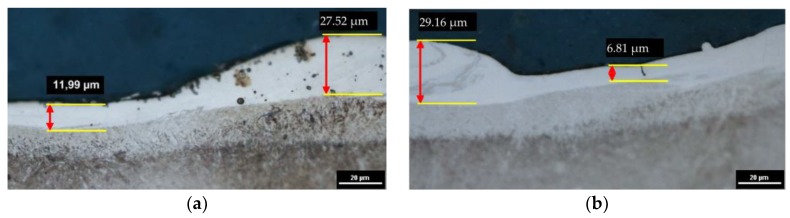
Themetallographic structure of 55NiCrMoV7 tool steel after EDM: (**a**,**b**) *Uc* = 25 V, *I* = 13.5 A, *t*_on_ = 145 µs, *t*_off_ = 50 µs; (**a**) pure dielectric and (**b**) dielectric with 0.1% RGO.

The recast layer is characterized by a high variability of thickness. The increased amount of eroded material corresponds to the increased volume of melted material that resolidified on the surface. An analysis of the metallographic structure after EDM shows that changing the properties of the medium in which discharges take place (from pure dielectric to dielectric with 0.1% RGO) significantly affects the thickness of the observed layers. In the case of parameters corresponding to the finishing EDM (Figure 6a,b), the maximum white layer thickness is about 6.24 μm and 4.56 μm for manufacturing in pure dielectric and 0.1% RGO, respectively. However, for EDM with RGO flakes, the thickness of the white layer is more uniform. Analysis of the metallographic structure for semifinishing EDM parameters (Figure 7a,b) indicates that the maximal thickness of the white layer (WL) is about 21 μm and 16 μm for manufacturing in pure dielectric and 0.1% RGO, respectively. In the case of roughing manufacturing, the maximal thickness of the white layer is similar (Figure 8). For roughing parameters, the minimal thickness of the white layer is about 7 μm for 0.1% RGO. An observed significant difference in the thickness of the white layer indicates a local increase of discharge energy. It can be caused by a local change in the properties of the dielectric (uneven “distribution” of RGO in the dielectric). An analysis of the metallographic structure indicates that using RGO flakes in the dielectric leads to a more uniform distribution of the recast layer on the surface. An explanation for this effect can be found in the high thermal conductivity of RGO flakes, which store heat energy during the discharge and give it back after discharge to the dielectric. The dielectric softly cools the molten material, which resolidifies on the surface of the workpiece.

For the medium and highest discharge current and time pulse (Figure 7 and Figure 8), a significant increase in the thickness of the layers in relation to the finishing EDM was obtained. An observation of the images also shows that, for larger values of discharge energy, the white layer thickness is uneven across the surface (Figure 8). This is the result of the uneven distribution of melted material that was not removed from the crater and resolidified on the surface. 

**Figure 9:**
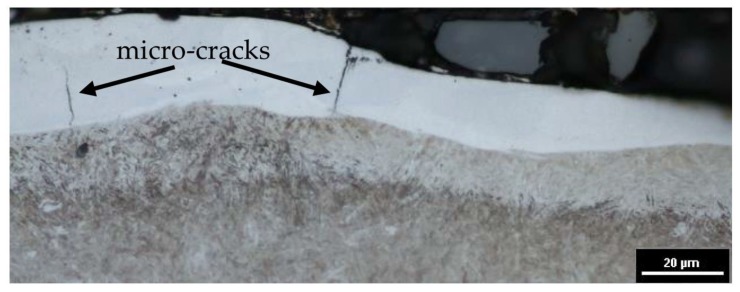
The metallographic structure of 55NiCrMoV7 tool steel after EDM.

As a result of interactions of thermal processes occurring during EDM, microcracks occur. The reason for their formation is thermal stresses. The molten material that was not ejected from the plasma channel and resolidified on the surface of the core has a much lower temperature. As a result of the cooling and resolidification of the molten layer, shrinkage occurs and tensile stresses are generated. Exceeding the maximum tensile strength of the material causes microcracks (Figure 9). Microcracks are an undesirable effect, causing, among other things, a reduction of fatigue strength and resistance to corrosion.

### 3.2. Surface Response Methodology

A central composite rotatable design with two factors and five levels was used for the experiment to establish the influence of discharge current and pulse time on roughness *Ra* and MRR during manufacturing in the pure dielectric and in the dielectric with 0.1% RGO. This type of experiment reduces the number of experimental runs required to generate sufficient information for a statistically adequate result. According to the central composite rotatable design, 10 samples with one replication in the center point were manufactured and measured. 

An investigation of the influence of EDM parameters on roughness *Ra* and MRR was carried out using response surface methodology (RSM). In RSM, a regression model is built to predict the influence of investigated parameters on independent variables. The choice of the function should consider the best fit of the experimental results for the response function. In this study, the second-degree polynomial function was used to fit the response function to the experimental results:

(3) $$Y=\beta_{0}+\sum_{i=1}^{k}\beta_{i}X_{i}+\sum_{i=1}^{k}\beta_{i}i\;X_{i}^{2}+\sum_{i=1,i<j}^{k}\beta_{i\;j}i\;X_{i}X_{j}+\varepsilon$$

An analysis of variance (ANOVA) was used to check the significance of the independent variable in the model. The ANOVA test was conducted at a 95% coefficient level. If the probability value (Prob > *f*) for the factor was less than 0.05, this indicated that the model factor was significant (i.e., at a 95% confidence level). A value of Prob > *f* higher than 0.05 indicated that the model factor was nonsignificant and should be removed from the response function. After removing nonsignificant factors, the ANOVA test was conducted again for a new function (without nonsignificant terms).

**Figure 10:**
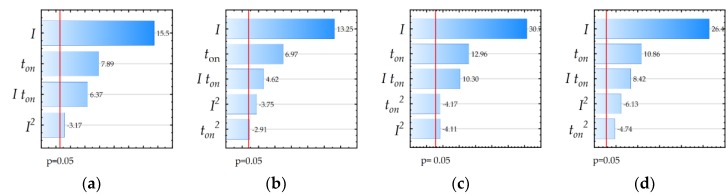
The Pareto chart of effects of significant factors in developed models: (**a**) *Ra* for EDM in the pure dielectric; (**b**) *Ra* for EDM with 0.1% RGO in the dielectric; (**c**) MRR for EDM in the pure dielectric; and (**d**) MRR for EDM with 0.1% RGO in the dielectric.

The ANOVA results for *Ra* and MRR are shown in Table 4, Table 5, Table 6 and Table 7. Table 4 and Table 5 show the results for the regression equation of roughness *Ra* for EDM in the pure dielectric and with 0.1% RGO, respectively. The calculated contributions indicate that the discharge current in both cases had the most influence on roughness *Ra* (68% and 65%). Second, the affecting variable was pulse time (about 18%). Other variables and their interactions had a significant influence on roughness *Ra*, but their contributions were lower. Table 6 and Table 7 present the ANOVA results of MRR for EDM in the pure dielectric and with 0.1% RGO, respectively. The calculated contributions indicate that the discharge current (75%) had the most influence on MRR, followed by pulse time (about 13%), for both cases of manufacturing. Other variables and their interactions were significant, but their contributions were lower. The Pareto chart (Figure 10) shows the absolute values of the standardized effects for all developed models from the largest to the smallest effect.

From the presented ANOVA (Table 4, Table 5, Table 6 and Table 7), the calculated Fisher coefficient for *Ra* was 17.68 and MRR was 13.43 for EDM in the pure dielectric. For EDM in the dielectric with 0.1% RGO, the Fisher coefficient for *Ra* and MRR was 62.61 and 47.31, respectively. The results implied that all the developed models were significant at the 95% confidence level.

The response function for investigated parameters was established by regression analysis. A backward elimination process was performed. For each response function, the coefficient of determination, *R-sqr*, and the adjusted coefficient of determination, *R-adj*, were calculated. These coefficients represent the percentage of variance explained by the model. For *R-sqr* and *R-adj* approaching unity, the response function is a more accurate fit for the research results.

After ANOVA testing, the response equations for roughness *Ra* and MRR can be described by the following polynomial function:

- For EDM in the pure dielectric
  *Ra* = 0.61 − 0.01 *t_on_* + 0.76 *I* − 0.033 *I*^2^ + 0.004 *I t_on_* (4)
  MRR = −3.23 + 0.038 *t_on_* − 0.0003 *t_on_*^2^ + 1.48 *I* − 0.049 *I*^2^ + 0.008 *I t_on_.* (5)
- For EDM in the dielectric with 0.1% RGO flakes
  *Ra* = −1.73 + 0.027 *t_on_* − 0.0002 *t_on_*^2^ − 0.78 *I* − 0.035 *I*^2^ + 0.003 *I t_on_* (6)
  MRR = −6.05 + 0.074 *t_on_* − 0.0006 *t_on_*^2^ + 2.69 *I* − 0.11 *I*^2^ + 0.01 *I t_on_*. (7)

**Figure 11:**
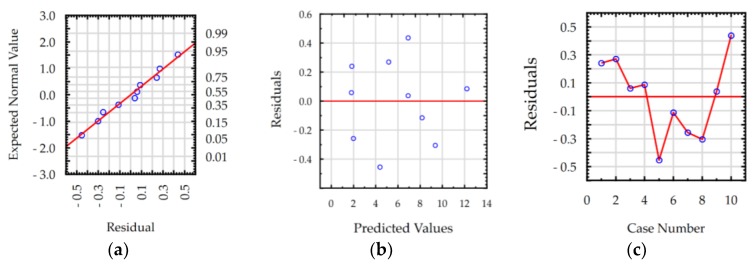
Plots of residuals for roughness *Ra* model, EDM in the pure dielectric: (**a**) normal plot of residuals, (**b**) residuals versus predicted values, and (**c**) residuals versus case number.

**Figure 12:**
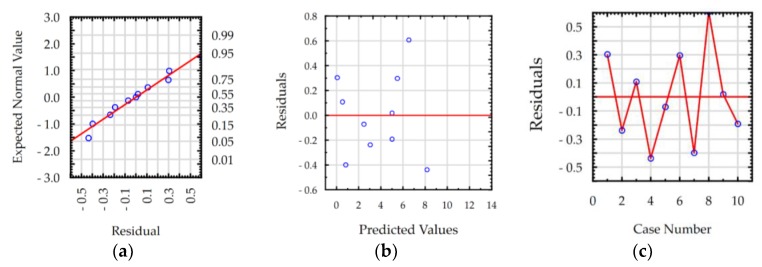
Plots of residuals for roughness *Ra* model, EDM in the dielectric with 0.1% RGO: (**a**) normal plot of residuals, (**b**) residuals versus predicted values, and (**c**) residuals versus case number.

**Figure 13:**
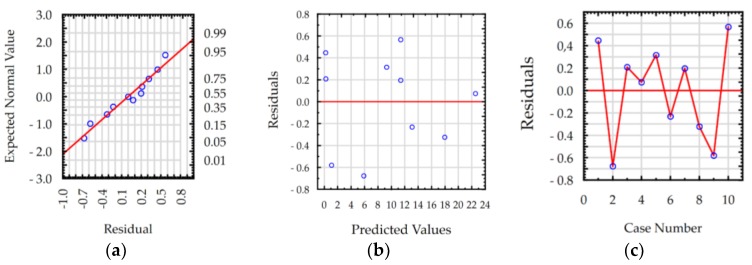
Plots of residuals for MRR model, EDM in the pure dielectric: (**a**) normal plot of residuals, (**b**) residuals versus predicted values, and (**c**) residuals versus case number.

**Figure 14:**
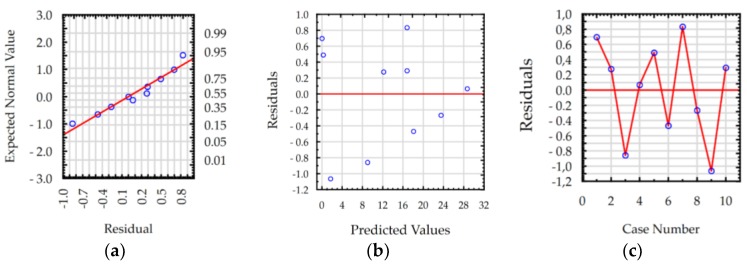
Plots of residuals for MRR model, EDM in the dielectric with 0.1% RGO: (**a**) normal plot of residuals, (**b**) residuals versus predicted values, and (**c**) residuals versus case number.

The ANOVA results show that the values of R-squared for roughness *Ra* and MRR were over 98% and 99%, respectively, for manufacturing in both the pure dielectric and with 0.1% RGO. The results indicate that the regression models provided an excellent explanation of the relationship between discharge current, pulse time, and response *Ra* and MRR. The difference between R-adjustable value relative to R-squared is smaller than 0.1, which indicates that the established models were adequate and represented the process. A residual analysis was performed to check the quality of fit of the response model roughness *Ra* and MRR with the experimental results. The normal probability plots of residuals (Figure 11a, Figure 12a, Figure 13a and Figure 14a) show that the experimental data are distributed approximately along a straight line. This shows that the correlation between predicted and experimental data is good. The plots of residuals versus predicted values (Figure 11b, Figure 12b, Figure 13b and Figure 14b) and residuals versus case number values (Figure 11c, Figure 12c, Figure 13c and Figure 14c) show that the residuals did not follow any trend and have a stochastic nature. The plotted residuals versus case number shows that the error terms were independent of one another. The analysis of the residual plots indicates that the developed models were adequate from a statistical point of view.

**Figure 15:**
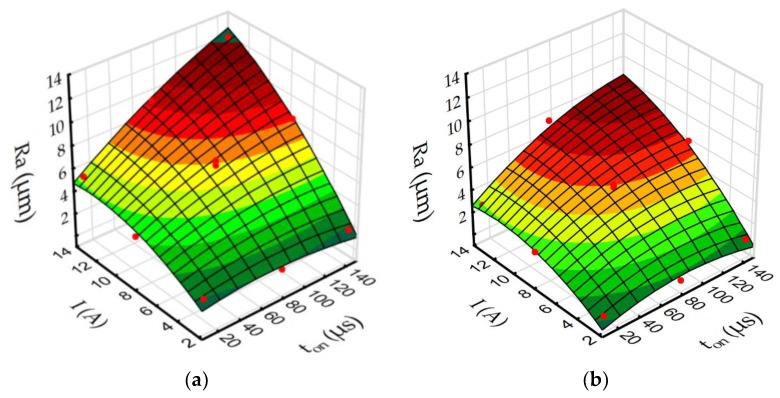
The estimated response surface plot for roughness *Ra*: (**a**) EDM in pure dielectric and (**b**) EDM in dielectric with 0.1% RGO.

**Figure 16:**
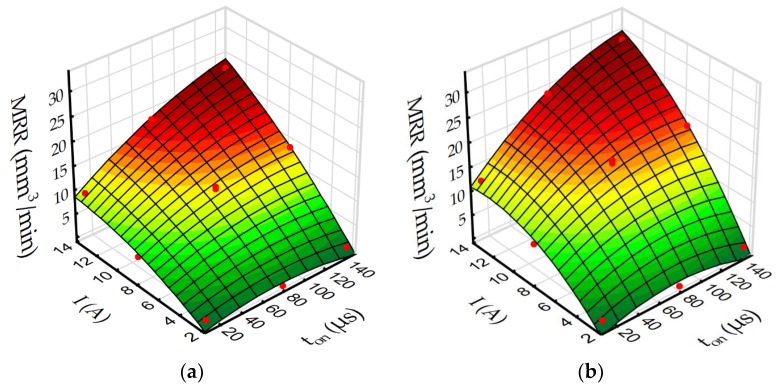
The estimated response surface plot for MRR: (**a**) EDM in pure dielectric and (**b**) EDM in dielectric with 0.1% RGO.

Based on the regression models (Equations (4)–(7)) to better understand the influence of discharge current and pulse time on roughness *Ra* and MRR during EDM in the pure dielectric and with 0.1% RGO, response surface plots were estimated (Figure 15 and Figure 16).

The results indicate that using reduced graphene oxide flakes in the dielectric has a strong influence on roughness *Ra* and MRR. In both cases (EDM in the pure dielectric and in the dielectric with 0.1% RGO) roughness *Ra* increases with the growth of the discharge current and the pulse time (Figure 15). For a constant voltage, discharge current and pulse time determine the amount of energy of the electrical discharge. At the lowest value of discharge current, increasing the time pulse does not increase the volume of material removed in discharge. This is related to the amount of heat flux delivered to the workpiece, which causes melting and evaporation of material. A comparison of Figure 15a,b shows that for the same discharge current and pulse time, using RGO flakes in the dielectric leads to a decrease of roughness *Ra*. This can be explained by different discharge propagation with the additional particles in the dielectric relative to conventional EDM. The presence of additional conductive particles in the dielectric reduced the breakdown voltage. Furthermore, additional particles can cause the occurrence of several bridges in which the plasma channel will be created. This leads to multiple discharges during one pulse [58]. Since the reduced graphene oxide flakes have a free vacant electron [59], they can accumulate the electrons and act as a capacitor. Releasing the electrons can lead to dispersion of discharge on another flake. These two effects cause a decrease in energy delivered to the workpiece. Generated craters have a smaller diameter and are shallow relative to manufacturing in the pure EDM. 

The changes in the propagation of the discharge also have an effect on MRR (Figure 16). The presence of RGO flakes in the dielectric reduces the electrical resistivity of the dielectric, which leads to discharge in the increasing gap size relative to conventional EDM. This leads to the easier flushing of the debris, and as a consequence, more stable discharges are obtained. Furthermore, the presence of reduced graphene oxide flakes in the dielectric leads to multiple discharges during one pulse. This leads to an increase of the frequency of discharge, which overcomes the decrease of removed volume material by reduced discharge energy. The use of additional conductive particles in the dielectric obtains smaller craters and smaller debris particles, which are removed from the larger gap, which accelerates MRR.

## 4. Conclusions

The experiments were focused on the influence of reduced graphene oxide flakes in the dielectric on electrical discharge propagation and heat dissipation in the gap during the machining of 55NiCrMoV7 tool steel. The results indicate that using RGO flakes in the dielectric leads to decreased surface roughness and thickness of the recast layer with increased MRR. The presence of RGO flakes in the dielectric reduced the breakdown voltage and caused several discharges to occur during one pulse. The dispersion of the discharges caused a decrease in the energy delivered to the workpiece.

On the basis of theoretical analyses and experimental research, the following conclusions are drawn:

- Changing the dielectric properties by adding RGO flakes in EDM has a significant effect on the surface topography. A reduction in the discharge energy by dispersion on RGO flakes leads to the generation of craters with a smaller diameter and depth compared with those produced by machining without RGO in the dielectric.
- The results show that by using 0.1% RGO flakes in the dielectric for finishing EDM parameters, it is possible to obtain a 460% reduction of roughness *Ra* with a slight increase in MRR (12%). In the case of roughing EDM, the roughness *Ra* decreased by 60% with a 27% increase in MRR.
- An analysis of the metallographic structure indicated that using RGO flakes in the dielectric leads to a more uniform distribution of the recast layer on the surface. RGO flakes store the heat energy during the discharge and give it back to the dielectric after the discharge. The dielectric softly cools the molten material, which resolidifies on the surface of the workpiece in a uniform manner.
- The presence of RGO flakes on the dielectric reduces the electrical resistivity, which leads to an increased gap size. The easier flushing of the debris leads to more stable discharge. Furthermore, multiple discharges during one pulse increased the frequency of discharge, which overcomes the decrease in removed volume material by reducing the discharge energy.

## Figures and Tables

**Table 1:** The chemical composition of 55NiCrMoV7 tool steel.

Chemical Composition (%)											
C	Mn	Si	P	S	Cr	Ni	Mo	W	V	Co	Cu
0.5–0.6	0.5–0.8	0.15–0.4	MAX 0.03	MAX 0.03	0.5–0.8	1.4–1.8	0.15–0.25	MAX 0.3	MAX 0.1	MAX 0.3	MAX 0.3

**Table 2:** The electrical discharge machining (EDM) conditions.

Electrode	Copper Cross Section 12 × 12 mm
Material	55NiCrMoV7 tool steel
Polarization of electrode	Positive polarity
Discharge current *I*_c_ (A)	1.7, 2, 8, 13.5, 14
Open voltage U_0_ (V)	225 V
Discharge voltage (V)	25
Pulse time *t*_on_ (μs)	5, 10, 75, 145, 150
Time interval *t*_off_ (μs)	0.3 *t*_on_
RGO in dielectric (%)	0, 0.1
Manufacturing depth (mm)	0.2

**Table 3:** The design of the experimental matrix with measured surface roughness parameters: Ra, roughness; MRR, material removal rate.

Ex. no.	EDM Parameters			Pure Dielectric		Dielectric with 0.1% RGO	
	Pulse Duration *t*_on_ (μs)	Discharge Current *I*_c_ (A)	Discharge Energy (mJ)	*Ra* (μm)	MRR (mm^3^/min)	*Ra* (μm)	MRR (mm^3^/min)
1	10	2	0.5	2.09	0.64	0.37	0.72
2	10	13.5	3.4	5.43	9.6	2.8	12.46
3	145	2	7.2	1.86	0.42	0.64	0.74
4	145	13.5	48	12.29	22.64	7.72	28.77
5	5	8	1	3.92	5.21	2.4	8.16
6	150	8	30	8.05	12.86	5.79	17.62
7	75	1.7	3.2	1.73	0.48	0.44	0.64
8	75	14	26.2	9.04	17.64	7.12	23.21
9	75	8	15	6.95	11.58	5.03	17.08
10	75	8	15	7.35	11.95	4.82	17.62

**Table 4:** The ANOVA table for *Ra* (EDM in the pure dielectric).

Source	Sum of Squares	Degrees of Freedom	Mean Square	*F*-Value	Prob > *f*	Contribution %
Model	109.404	4	27.351	17.680	<0.0001	
*t_on_*	19.288	1	19.288	62.31	0.0005	17.63
*I*	74.425	1	74.425	240.44	<0.0001	68.03
*I* ^2^	3.113	1	3.113	10.06	0.0247	2.85
*I t_on_*	12.578	1	12.578	40.64	0.0014	11.50
Error	1.547	5	0.309			
Total SS	110.951	9	*R-sqr* = 0.98		*R-adj* = 0.97	

**Table 5:** ANOVA table for *Ra* (EDM with 0.1% RGO in the dielectric).

Source	Sum of Squares	Degrees of Freedom	Mean Square	*F*-Value	Prob > *f*	Contribution %
Model	67.8361	5	13.567	13.432	<0.0001	
*t_on_*	12.298	1	12.298	48.67	0.0022	18.13
*t_on_* ^2^	2.1401	1	2.140	8.46	0.0436	3.15
*I*	44.421	1	44.421	175.79	0.0001	65.48
*I* ^2^	3.564	1	3.564	14.10	0.0198	5.25
*I t_on_*	5.413	1	5.413	21.42	0.0098	7.98
Error	1.010	4	0.252			
Total SS	68.82661	9	*R-sqr* = 0.98		*R-adj* = 0.96	

**Table 6:** The ANOVA table for MRR (EDM in the pure dielectric).

Source	Sum of Squares	Degrees of Freedom	Mean Square	*F*-Value	Prob > *f*	Contribution %
Model	519.063	5	103.812	62.6131	<0.0001	
*t_o_*	69.715	1	69.715	168.16	0.0002	13.43
*t_on_* ^2^	7.242	1	7.242	17.47	0.0139	1.40
*I*	391.122	1	391.122	943.42	<0.0001	75.35
*I* ^2^	6.999	1	6.999	16.88	0.0147	1.35
*I t_on_*	43.985	1	43.985	106.09	0.0005	8.47
Error	1.658	4	0.414			
Total SS	520.721	9	*R-sqr* = 0.99		*R-adj* = 0.99	

**Table 7:** The ANOVA table for MRR (EDM with 0.1% RGO in the dielectric).

Source	Sum of Squares	Degrees of Freedom	Mean Square	*F*-Value	Prob > *f*	Contribution %
Model	885.6370	5	177.1274	47.309	<0.0001	
*t_on_*	110.568	1	110.568	118.124	0.0004	12.48
*t_on_* ^2^	21.083	1	21.083	22.524	0.0089	2.38
*I*	652.421	1	652.421	697.001	<0.0001	73.67
*I* ^2^	35.193	1	35.193	37.598	0.0035	3.97
*I t_on_*	66.372	1	66.372	70.908	0.0010	7.49
Error	3.744	4	0.936			
Total SS	889.381	9	*R-sqr* = 0.99		*R-adj* = 0.99	
